# Factors associated with contralateral liver hypertrophy after unilateral radioembolization for hepatocellular carcinoma

**DOI:** 10.1371/journal.pone.0181488

**Published:** 2017-07-14

**Authors:** Juliane Goebel, Maximilian Sulke, Andrea Lazik-Palm, Thomas Goebel, Alexander Dechêne, Alexander Bellendorf, Stefan Mueller, Lale Umutlu, Jens Theysohn

**Affiliations:** 1 Department of Diagnostic and Interventional Radiology, University Hospital Essen, Essen, Germany; 2 Clinic for Gastroenterology, Hepatology and Diabetology, Petrus Hospital, Wuppertal, Germany; 3 Clinic for Gastroenterology and Hepatology, University Hospital Essen, Essen, Germany; 4 Clinic for Nuclear Medicine, University Hospital Essen, Essen, Germany; Texas A&M University, UNITED STATES

## Abstract

**Introduction:**

Radioembolization for the treatment of hepatocellular carcinoma (HCC) induces liver volume changes referred to as “atrophy-hypertrophy complex”. The aim of this study was to investigate lobar liver volume changes after unilateral radioembolization and to search for factors associated with hypertrophy of the untreated lobe.

**Materials and methods:**

Seventy-five patients were retrospectively evaluated. Inclusion criteria were: (1) right-lobar radioembolization for unresectable unilateral HCC, (2) available liver computed tomography scans before, 1, 3, and at least 6 months after radioembolization. Baseline patient characteristics included clinical features, laboratory results, spleen volume, and liver computed tomography. Absolute and relative (referred to the whole liver volume) liver lobe volumes (LLV) as well as relative LLV (rLLV) change per month were evaluated and compared.

**Results:**

Absolute and relative contralateral LLV continuously increased after radioembolization (p<0.001). Mean relative contralateral LLV increased from 36±11.6% before radioembolization to 50±15.3% 6 months after radioembolization. Median contralateral rLLV increase/month (within first 6 months) was 2.5%. Contralateral rLLV increase/month was significantly lower in patients with ascites (p = 0.017) or platelet count <100/nl (p = 0.009). An inverse correlation of contralateral rLVV increase/month with spleen volume (p = 0.017), patient age (p = 0.024), Child Pugh score (p = 0.001), and tumor burden (p = 0.001) was found.

**Conclusions:**

Significant contralateral hypertrophy and ipsilateral atrophy were common after unilateral radioembolization. Small spleen volume, low patient age, low Child Pugh score, absence of ascites, platelet count ≥100/nl, and low tumor burden were associated with increased contralateral hypertrophy, indicating that younger patients with compensated cirrhosis might benefit most from radioembolization in a “bridge-to-resection” setting.

## Introduction

Radioembolization (RE) using Yttrium-90 (^90^Y) microspheres is an established treatment option in hepatocellular carcinoma (HCC) not curatively treatable by surgery or transplantation and is usually considered for patients with intermediate (Barcelona clinic liver cancer (BCLC) B) or advanced (BCLC C) tumor stages [1–3]. If certain precautions are met, RE is safe and effective. It combines the therapeutic effects of interstitial radiotherapy and to a lesser extent arterial microembolisation [4,5] and is known to induce liver volume changes referred to as “atrophy-hypertrophy complex” with atrophy of the treated and hypertrophy of the untreated liver lobe [6]. In unilobar HCC the hypertrophy of the contralateral lobe is of special interest as it represents the future liver remnant (FLR) if subsequent curative hemihepatectomy is considered [7]. In patients primarily not suitable for curative liver surgery due to small FLR, portal vein embolization is an option to induce contralateral lobar hypertrophy in order to achieve liver resection in a second step [8,9]. Some investigators have described faster HCC growth rates and an increased rate of contralateral metastasis in the formerly tumor-naïve lobe after portal vein embolization [10]. RE seems a promising alternative because it combines locoregional tumor therapy and induction of hypertrophy of the tumor-naïve contralateral liver lobe [7,11].

Only few studies have dealt with the extent of liver volume changes after unilateral RE in HCC patients so far. Up to now the factors influencing the extent of contralateral liver hypertrophy after RE are not identified. The aim of this study was to evaluate lobar liver volume changes after unilateral RE and to search for clinical parameters predictive of hypertrophy of the untreated lobe.

## Materials and methods

### Patients

All data were fully anonymized before access by the researchers, and retrospective analysis and use of data was approved by the local ethic committee (of the medical faculty, University Duisburg-Essen, Germany). Study inclusion criteria were: (A) right lobar RE for unresectable right unilobar HCC, and (B) computed tomography (CT) imaging of the liver available before, as well as 1, 3, and at least 6 months after RE. Exclusion criteria were: (C) previous surgical hepatic resection/segmentectomy and (D) transarterial chemoembolization within three months before or within six months after RE. A follow-up period of at least 6 months was chosen because volumetric lobar liver changes after unilateral RE are most pronounced in the first 6 months as described previously [12]. Baseline characteristics of patients including laboratory results and survival data were taken by chart review.

### Computed tomography of the liver/upper abdomen

Informed consent for contrast-enhanced liver CTs was given by all patients. Liver CTs were acquired in each patient before and 1, 3, and 6 months after RE. In 61 patients, an additional 9-months-follow-up liver CT was available. All CTs were conducted using a Somatom Definition scanner (Siemens Healthcare GmbH, Erlangen, Germany). Collimation was 128x0.6mm and images were acquired at approximately 120kVp utilizing Care kV^™^ and Care Dose4D^™^ (Siemens Healthcare GmbH, Erlangen, Germany) as dose-saving protocol. Automatic bolus triggering was used for arterial phase acquisition, placing the trigger in the descending aorta at the level of the celiac trunk. The portal venous phase of the abdomen was acquired 85 seconds after injection of the contrast medium. 100ml Ultravist 300 (Iopromid 300, Bayer HealthCare, Leverkusen, Germany) at a rate of 3.0ml/sec was administered, followed by 40ml saline at the same rate. Images were reconstructed using B30f kernel in 5mm slice thickness.

### Radioembolization (RE)

Preparation for RE was performed by digital subtraction angiography of the liver including 99m-technetium labelled human serum albumin microspheres scan (^99m^Tc-HSAM, B20, ROTOP Pharmaka AG, Dresden, Germany) and calculation of hepato-pulmonary shunt fraction [13]. Starting with a selective digital subtraction angiography of the celiac trunk and the superior mesenteric artery, the position (or positions, depending on the hepatic arterial tree) for ^99m^Tc-HSAM administration was defined aiming on selective right liver lobe coverage. The mean lung shunt fraction was 5.7±3.3% (range = 1–14%). Consecutive right lobar RE was conducted in the same catheter position as the preparation digital subtraction angiography with the administration of the ^90^Y glass microspheres (TheraSphere^™^, BTG, Hertfordshire, United Kingdom). On average 2.9±1.2GBq (range = 1.2–7.3GBq) ^90^Y were administered, resulting in a mean right liver lobe dosage of 113±11Gy (range = 70-150Gy).

### Assessment of baseline liver findings and volumetry

All baseline CTs were evaluated for the presence of ascites, portocaval shunts, and portal vein thrombosis by an experienced radiologist (>5 years of experience in abdominal imaging). The majority of HCC patients suffered from multifocal right lobar disease, therefore no exact tumor burden volumetry was feasible and the right liver lobe tumor burden was assessed categorially (0–25%, 26–50%, 51–75%, 76–100%).

Baseline spleen volume and lobar liver volumes (LLV) for each examination were assessed by manual drawing of regions of interest (ROI) in every second 5mm axial CT slice. Consecutively all ROIs were summed up and multiplied by 10mm for volume calculation. The absolute right LLV was defined as the volume of Couinaud segments 5 to 8 [14], absolute left LLV as the volume of segments 1 to 4. The relative LLV (rLLV) was defined as absolute LLV/whole liver volume and is given in %. The left rLLV was used as a surrogate for the FLR (in hypothetical subsequent right hepatic lobectomy). rLLV changes per month were calculated by the following formula: rLLV changes/month = (rLLV (6 months after RE)–rLLV (before RE))/6 months.

### Statistical analysis

For statistical analysis SPSS Statistics 19.0 (IBM Corporation, Armonk, NY, USA) and MedCalc 15.6 (MedCalc Software, Ostend, Belgium) were used. Data were tested for normal distribution using D’Agostino Pearson test. Normal distributed data are presented as mean, standard deviation, and range. Not normally distributed data are presented as median and range. Association between patient age, stage of cirrhosis, and right lobar tumor burden was analyzed via Spearman correlation analysis. Survival analysis was expressed by Kaplan Meier statistics. Test for mean/median differences of absolute/relative LLV between follow-up time points was performed using Wilcoxon test or paired t test, as appropriate. Tests for differences of baseline rLLV and rLLV changes/month depending on baseline patient characteristics were performed using univariate analysis of variance, unpaired t test, Spearman correlation analysis, and stepwise linear regression analysis, as appropriate. A p-value <0.05 was considered statistically significant.

## Results

### Patients including baseline characteristics

**Fig 1:**
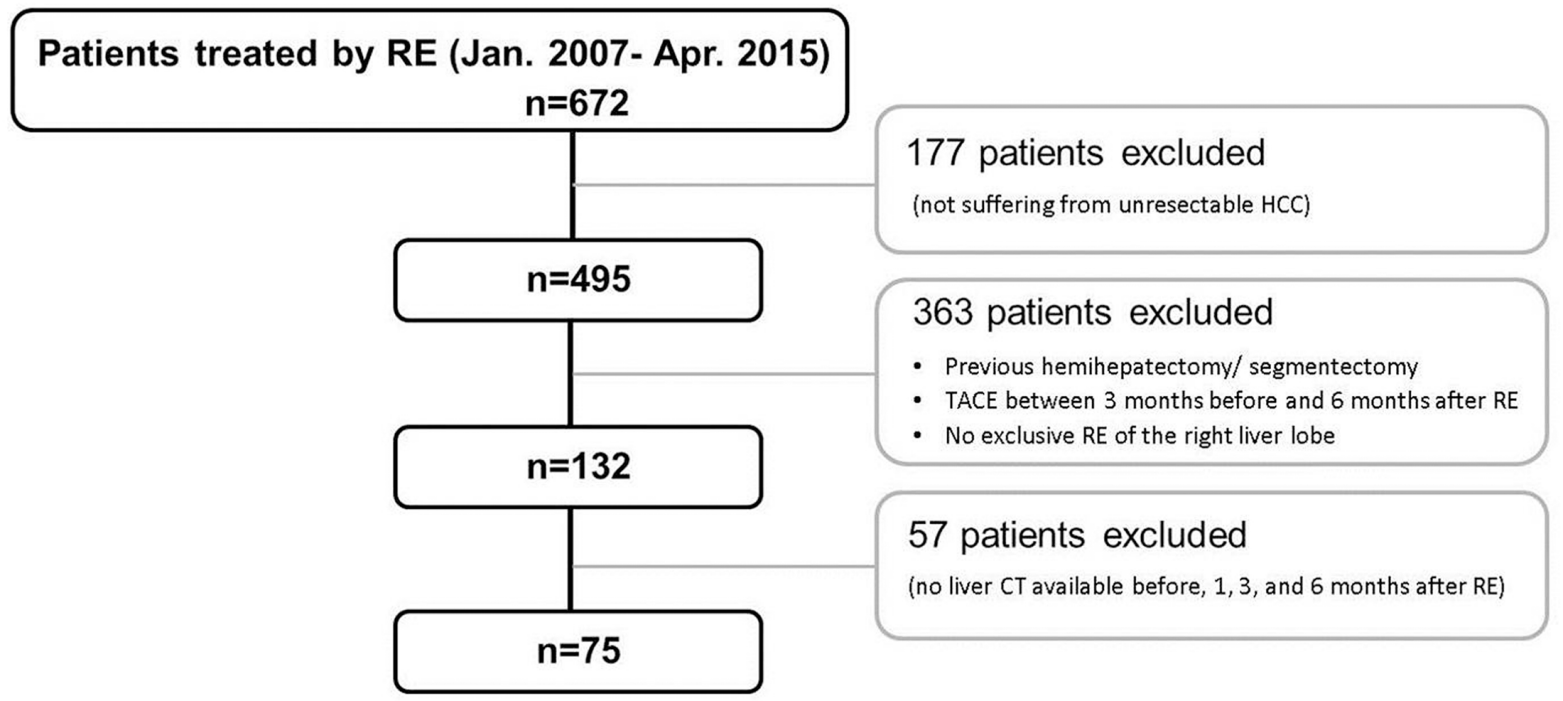
Study flow chart. From 672 patients treated by RE between January 2007 and April 2015 at the study center only 75 fitted the current inclusion and exclusion criteria.

Six hundred and seventy-two patients were treated by RE between January 2007 and April 2015 at our center (509 males; 163 females; mean age = 64.9±11.5 years; Fig 1). Only 495 of them suffered from HCC. One hundred and fifty-three patients suffered from hepatic metastases (mainly of neuroendocrine tumors (42.5%), colorectal cancers (30.1%), and uveal melanomas (5.9%)), 17 patients from cholangiocellular carcinoma, four patients from mixed HCC/cholangiocellular carcinoma, two patients from hepatic hemangiosarcoma, and one from hepatic hemangioendothelioma. Finally, out of the 495 patients treated by RE for HCC only 75 met all study inclusion and exclusion criteria (60 males; 15 females; mean age = 66.8±9.1 years) due to insufficient CT follow-up, previous hemihepatectomy or segmentectomy, TACE between 3 months before and 6 months after RE, and no exclusively right-sided RE (Fig 1).

The baseline characteristics of the included 75 patients are presented in Table 1. In cirrhotic patients the median Child Pugh Score was 5, ranging from 5 to 7. In 7 patients without liver cirrhosis, no liver disease was found, the other patients suffered from alcoholic liver disease (n = 14), chronic hepatitis B (n = 12), chronic hepatitis C (n = 13), non-alcoholic steatohepatitis (n = 22), secondary biliary cirrhosis (n = 1), and cryptogenic liver cirrhosis (n = 6). In the present cohort patient age was directly correlated with right tumor burden (r = 0.36; p-value = 0.002) and indirectly correlated with Child Pugh class (no cirrhosis; Child Pugh A; Child Pugh B; r = -0.24; p-value = 0.038).

**Table 1:** Patient baseline characteristics.

Laboratory results		
	Mean ± SD	Range
International Normalized Ratio	1.1 ± 0.1	0.9 to 1.4
Albumin [g/dl]	4.0 ± 0.5	1.2 to 4.9
Platelet count [/nl]	162 ± 92	39 to 500
Total bilirubin [mg/dl]	1.0 ± 0.5	0.2 to 2.0
Gamma-glutamyltransferase [U/l]	216 ± 300	30 to 2383
Tumor burden (right liver lobe) [absolute number (in %)]		
0–25%	48 (64%)	
26–50%	15 (20%)	
51–75%	9 (12%)	
76–100%	3 (4%)	
Ascites [absolute number of patients (in %)]		
Present	21 (28%)	
Absent	54 (72%)	
Portocaval shunts [absolute number of patients (in %)]		
Present	24 (32%)	
Absent	51 (68%)	
Portal vein thrombosis [absolute number of patients (in %)]		
Main trunk	0 (0%)	
Right branch	6 (8%)	
Left branch	2 (3%)	
Absent	67 (89%)	
Cirrhosis/Child Pugh class [absolute number of patients (in %)]		
No cirrhosis	20 (27%)	
Child Pugh A	46 (61%)	
Child Pugh B	9 (12%)	
Child Pugh C	0 (0%)	

SD, standard deviation.

### Survival

Mean survival after RE of all patients was 31±3.4 months. Stratification by presence or absence of cirrhosis and Child Pugh class (no cirrhosis, 28±3.7 months; Child Pugh A, 32±4.9 months; Child Pugh B, 27±5.7 months; p-value not significant (ns)) did not reveal significant differences in the mean survival. Stratification by the four tumor burden categories (0–25% tumor burden of right liver lobe, 35±4.6 months; 26–50% tumor burden, 25±5.7 months; 51–75% tumor burden, 20±2.8 months; 76–100% tumor burden, 18±2.3 months; p-value ns) did not show significant differences in the mean survival, but stratification by a tumor burden of 0–25% versus >25% did (0–25% tumor burden, 35±4.6 months; 26–100% tumor burden, 22±3.4 months; p-value = 0.042).

### Volumetric baseline values and volumetric changes

**Fig 2:**
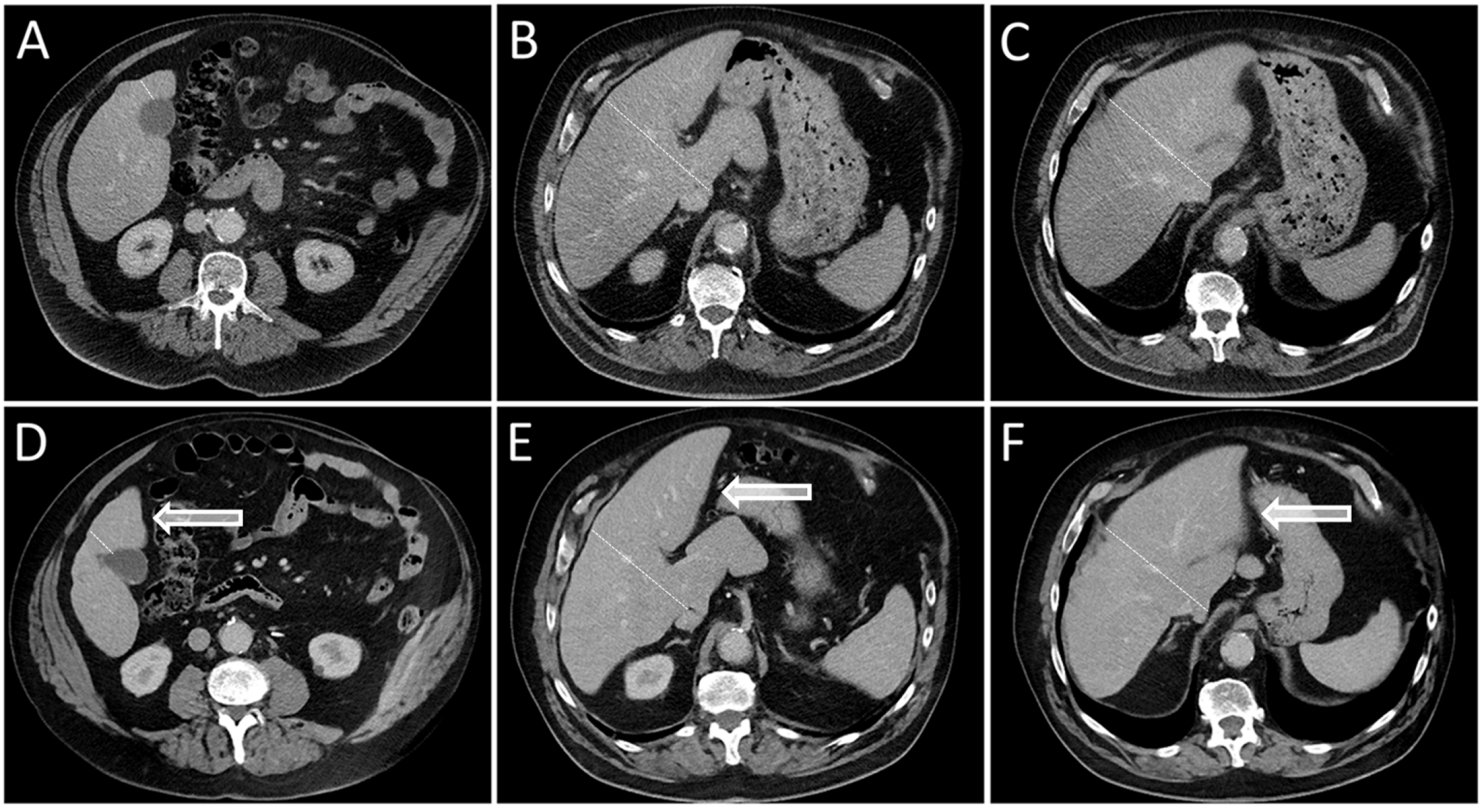
Example of left lobar hypertrophy after right-sided RE. Liver computed tomography images acquired in three different levels before RE (A-C) and 6 months after right-sided RE (D-F) show left lobar hypertrophy (highlighted by arrows) and right lobar atrophy in a 65-year-old man suffering from HCC and non-alcoholic steatohepatitis. (The liver lobe border is marked by a dotted line.).

**Fig 3:**
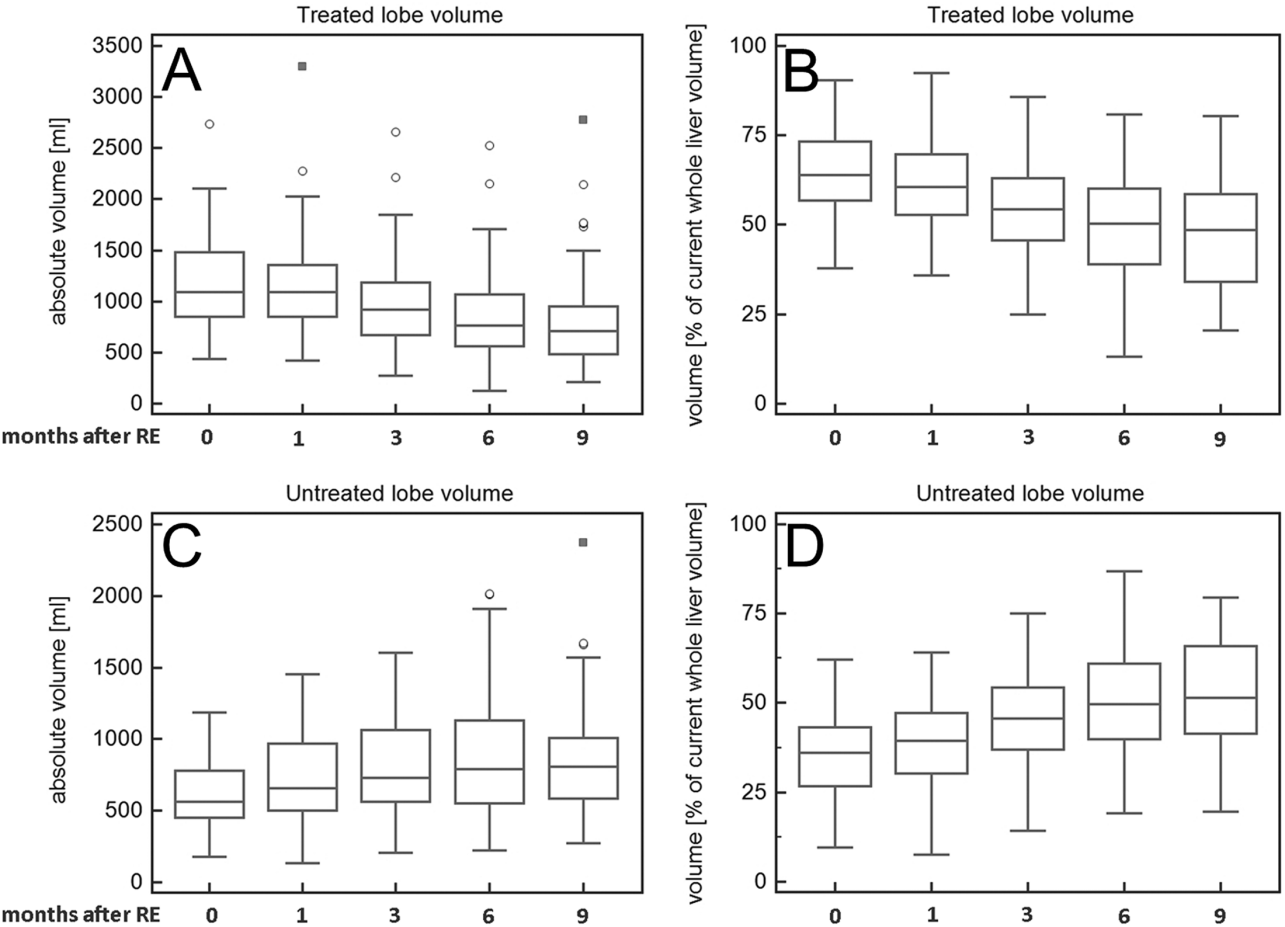
Volume changes after unilobar RE illustrated by box and whisker plots. Absolute and relative volume of the treated right liver lobe decreased (A, B) and absolute and relative volume of the untreated left liver lobe increased (C, D) after right-sided RE. (“Outside” values are represented as circles and “far out” values are represented as squares.).

**Fig 4:**
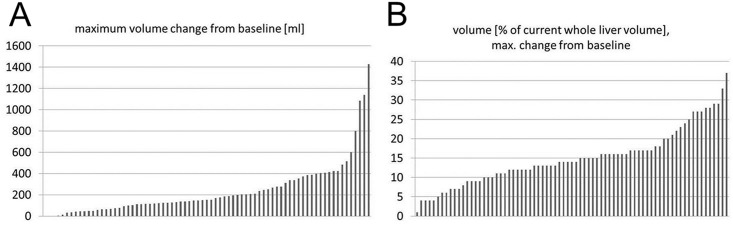
Maximum contralateral lobar volume increase after right-sided RE. Waterfall plots illustrating the maximum absolute (A) and relative (B) volume increase of the untreated left liver lobe within 6 months after RE in all patients.

Median baseline spleen volume was 392ml (range = 79-1646ml). Median baseline absolute right LLV was 1094ml (range = 433-2737ml) and median baseline left LLV was 562ml (range = 176-1187ml, Table 2). A continuous decrease of absolute right LLV was found after RE (Table 2, Figs 2 and 3) which became significant after more than one month after RE (Table 2). The absolute left LLV increased significantly at each time point in the follow-up after RE, and left LLV increase was significant at each follow-up time (Table 2). Accordingly, the right rLLV decreased and the left rLLV increased after RE (Table 2, Figs 2, 3 and 4). All rLLV changes were significant (Table 2). The mean left rLLV increased from 36±11.6% at baseline to 50±15.3% 6 months after RE and (in the 61 patients with follow-up at 9 months) to 53±16.1% 9 months after RE. Within the first 6 months after RE, the median decrease of right LLV/month was -52ml (range = -142-66ml), the mean decrease of right rLLV/month was -2.5±1.3%. The median increase of left LLV/month (within first 6 months after RE) was 26ml (range = -15-208ml), the mean increase of left rLLV/month was 2.5±1.3%.

**Table 2:** Absolute (presented as median and range) and relative volumetric liver lobe changes (presented as mean, standard deviation, and range) after RE.

Time after radioembolization	Absolute volume [ml]		Relative volume [% of whole liver volume]	
	Right lobe	Left lobe	Right lobe	Left lobe
0 month (n = 75)	1094 (433 to 2737)	562 (176 to 1187)	64 ± 11.6 (38 to 90)	36 ± 11.6 (10 to 62)
p-value (0 vs. 1 month)	0.130	<0.001	<0.001	<0.001
1 month (n = 75)	1091 (420 to 3301)	657* (136 to 1453)	61 ± 12.2* (36 to 92)	39 ± 12.2* (8 to 64)
p-value (1 vs. 3 months)	<0.001	<0.001	<0.001	<0.001
3 months (n = 75)	917* (276 to 2660)	727* (205 to 1602)	55 ± 13.6* (25 to 86)	45 ± 13.6* (14 to 75)
p-value (3 vs. 6 months)	<0.001	<0.001	<0.001	<0.001
6 months (n = 75)	765* (126 to 2526)	792* (221 to 2018)	50 ± 15.3* (13 to 81)	50 ± 15.3* (19 to 87)
p-value (6 vs. 9 months)	0.006	<0.001	<0.001	<0.001
9 months (n = 61)	713* (214 to 2778)	806* (274 to 2373)	47 ± 16.1* (21 to 80)	53 ± 16.1* (20 to 79)

N, number; vs., versus;

*, significant volumetric change in comparison to the preliminary computed tomography.

### Factors associated with baseline relative left lobar liver volume

Baseline left rLLV was significantly higher in patients suffering from cirrhosis (38±12.1%) than in patients without cirrhosis (30±8.0%; p-value = 0.002, Table 3). Baseline spleen volume and baseline left rLLV correlated significantly (r = 0.23; p-value = 0.049). A tendency towards a higher baseline of left rLLV was found in patients with thrombosis of the right main portal vein branch (n = 6; 44±14.4%) in comparison to patients without portal vein thrombosis (n = 67; 35±11.2%; p-value = 0.055).

**Table 3:** Factors associated with baseline relative left lobar liver volume and its change after RE.

			r	p value
Baseline relative left lobar liver volume				
Cirrhosis	Yes	38 ± 12.1%		0.002
	No	30 ± 8.0%		
Right portal vein branch thrombosis	Yes	44 ± 14.4%		0.055
	No	35 ± 11.2%		
Spleen volume			0.23	0.049
Relative left lobar liver volume increase/month after RE				
Ascites	Yes	1.9 ± 1.3%/month		0.017
	No	2.7 ± 1.3%/month		
Platelet count <100/nl	Yes	1.9 ± 1.1%/month		0.009
	No	2.8 ± 1.4%/month		
Child Pugh score			-0.42	0.001
Right lobar tumor burden			-0.36	0.001
Spleen volume			-0.27	0.017
Patient age			-0.26	0.024
Ascites			-0.25	0.032
Portal vein thrombosis			-0.21	0.078
Portocaval shunting			-0.06	0.632

R, correlation coefficient.

### Factors influencing relative liver volume changes after radioembolization

**Fig 5:**
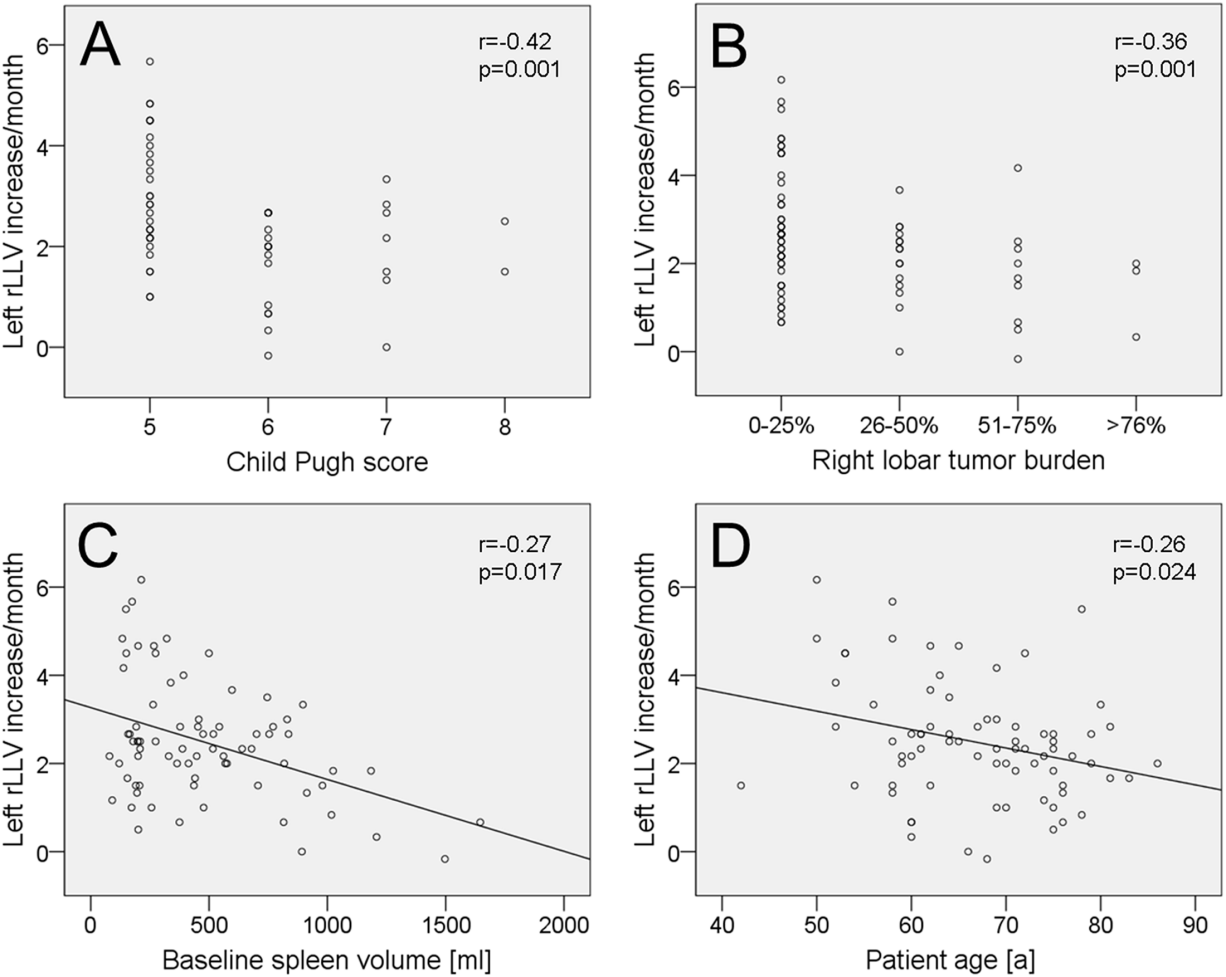
Factors influencing relative left liver volume change after right lobar RE. Scatterplots demonstrating the correlation between relative left liver volume (rLLV) change after RE [%/month] and Child Pugh score (A), right lobar tumor burden (B), spleen volume (C), and patient age (D).

Left rLLV increase/month (within 6 months after RE) was significantly higher in patients without ascites (2.7±1.3%/month) in comparison to patients presenting with ascites (1.9±1.3%/month; p-value = 0.017, Table 3). Left rLLV/month was significantly higher in patients with a platelet count ≥100/nl (2.8±1.4%/month) compared to patients with a platelet count <100/nl (1.9±1.1%/month; p-value = 0.009). Left rLLV increase/month was inversely correlated with Child Pugh score (in patients with liver cirrhosis; r = -0.42; p-value = 0.001, Fig 5), right lobar tumor burden (r = -0.36; p-value = 0.001), baseline spleen volume (r = -0.27; p-value = 0.017), patient age (r = -0.26; p-value = 0.024), and presence of ascites (r = -0.25; p-value = 0.032). Only a trend towards correlation was found for left rLLV increase/month and the presence of portal vein thrombosis at baseline (r = -0.21; p-value = 0.078). No significant correlation was detected between left LLV increase/month and presence of portocaval shunting. Stepwise linear regression analysis revealed that baseline spleen volume was the most important factor for left rLLV increase/month prediction (r = -0.01; p-value = 0.001; beta = -0.483), followed by patient age (r = -0.26; p-value = 0.009; beta = -0.289) and Child Pugh score (r = -1.956; p-value = 0.044; beta = -0.233).

## Discussion

In the present study of 75 patients, suffering from HCC limited to the right liver lobe, we found significant contralateral hypertrophy and ipsilateral atrophy after right unilobar RE. The untreated left rLLV increased from 36±11.6% before RE to 50±15.3% 6 months after RE. Factors associated with contralateral lobar hypertrophy were lower spleen volume, low patient age, low Child Pugh score, absence of ascites, platelet count ≥100/nl, and low tumor burden.

Similar volumetric changes after RE were found by Edeline et al. in 34 HCC patients after either left or right lobar RE [15,16]. Edeline et al. reported a mean relative increase of 29% of the untreated LLV 3 months after RE which matches our results and which is comparable to the previously reported extent of contralateral hypertrophy after portal vein embolization [15,16]. The LLV of the treated lobe decreased 23% 3 months after RE in Edeline’s study which matches our results (decrease of 16%), too. Edeline et al. found, that baseline volumetric liver parameters (relative and absolute contralateral/untreated liver volume, absolute treated liver volume), tumor size, and platelet count influenced the degree of contralateral LLV increase. Apart from these factors Edeline et al. could not identify further parameters influencing contralateral liver hypertrophy which might be caused by the small study cohort.

In another study of 17 HCC patients treated with unilateral right RE, Gabe et al. described an even higher rLLV increase of 40% in the untreated lobe and a rLLV decrease of 52% in the treated lobe [17]. In comparison to Edeline et al., these higher volumetric changes might be caused by the extended follow-up with an average of 18 months. In our study, 6 months after RE a comparable contralateral rLLV hypertrophy of 41% and an ipsilateral rLLV atrophy of -30% were seen.

Vouche et al. reported on 83 patients treated with unilateral RE of which 67 suffered from HCC [7]. Three to six months after RE the left LLV had increased from 371ml to 505ml (+36%), matching our finding of a left LLV increase of +41%. Vouche et al. identified the presence of portal vein thrombosis as the single significant predictor of increase of FLR, which they defined in their study as consisting of Couinaud segments 2 and 3. Vouche et al. did not find any correlation of FLR increase and patient age or Child Pugh score. The disparity, regarding identifiable predictors of FLR increase, between Vouches and our findings might be caused by a different statistical approach: Vouche et al. used the relative FLR increase from baseline, whereas we used the rate of relative FLR increase from baseline per follow-up time. We chose our approach because the “atrophy-hypertrophy complex” previously was described as a linear time-dependent process [7]. Thus, the relative FLR increase/follow-up time more likely reflects the regenerative potential of the liver and might be more suitable for identification of predictors of FLR hypertrophy.

Liver regeneration after injury is mediated by parenchymal and non-parenchymal cells and regulated by several factors such as hyperplasia of the remnant hepatocytes [18], activation of myofibroblasts and hepatic stellate cells [19], production and release of local growth factors, chemokines and cytokines facilitating oval cell transformation which are assumed to represent liver stem/progenitor cells [20]. Hypertrophy of the untreated liver lobe in our study cohort was reduced in patients who suffered from splenomegaly, advanced or decompensated liver disease with ascites, or thrombocytopenia. The absence of relevant regeneration in decompensated cirrhosis is also seen in chronic viral hepatitis C infection, where in some patients the liver function does not sufficiently recover even after achieving sustained virological response [21]. This supports the concept of a “point of no return” beyond which the liver does not recover anymore. The main reason for this observation might be extensive fibrosis, which inhibits hyperplasia of hepatocytes, impairs so called stem-cell niches, and prevents stem cell activation [22,23].

The “atrophy-hypertrophy complex” is interpreted as uniform regenerative response of the liver to any type of damage [6]. After RE, radiation-induced liver disease occurs within weeks, prompts regenerative processes as mentioned above and clinically induces atrophy of the treated and hypertrophy of the untreated lobe. Moreover, some studies have described the development of portal hypertension after RE and the associated changes in portal venous blood flow might as well contribute to contralateral lobar hypertrophy [5,24]. The regenerative potential of the liver mainly depends on the quantity and quality of remnant liver tissue and is obviously reduced in liver cirrhosis. To the best of our knowledge this is the first study illustrating this in the “atrophy-hypertrophy complex” context after unilateral RE as higher Child Pugh scores, ascites, clinically obvious portal hypertension, assessed based on the surrogate criteria platelet count of <100/nl and splenomegaly [25], are significantly correlated with a decreased contralateral hypertrophy.

Not of less interest, in the present study hypertrophy of the untreated lobe is correlated with lower patient age. Liver regeneration is generally reduced in older patients [26] negatively affecting the outcome of unilobar RE in older patients.

Because not all patients equally benefit from a given treatment, it is of great clinical interest to select the “suitable” patients who will benefit most from RE. According to the mentioned parameters influencing contralateral liver hypertrophy, especially younger patients without obvious portal hypertension and with low Child Pugh scores may show pronounced contralateral liver hypertrophy, rendering them excellent candidates for a possible “bridge-to-resection” (or „hypertrophy-to-resection“) setting. This concept of adequate patient selection is currently also the basis of the stepwise therapy associating liver partition and portal vein ligation for staged hepatectomy (ALPPS). Because after an international registry showed a higher mortality after ALPPS in patients aged above 61 years [27], now this procedure is recommended only for selected, e.g. young patients [28]. In case of a unilateral HCC and initially too small FLR, especially in younger patients with low Child Pugh score RE should be considered as an alternative option to control local tumor and induce sufficient contralateral hypertrophy, rendering the patient suitable for subsequent curative surgical therapy in exceptional cases.

The current study is not without limitations. First and foremost, the number of reported patients is limited, but to our knowledge, this is the first study in HCC patients after RE evaluating the rate of relative FLR increase. Secondly, patients with Child Pugh A cirrhosis are overrepresented. That is due to the selection criteria for RE at our center and to the fact that only HCC patients were investigated. A precise conclusion for patients with higher Child Pugh scores can therefore not be drawn.

## Conclusions

In summary, significant contralateral lobar hypertrophy and ipsilateral lobar atrophy were seen after unilateral RE. Small spleen volume, low patient age, low Child Pugh score, absence of ascites, a platelet count ≥100/nl, and low tumor burden were associated with higher contralateral FLR hypertrophy, indicating that especially younger patients with compensated cirrhosis might benefit most from RE in the “bridge-to-resection” setting.

## Supporting information

S1 Table Raw data of patient baseline characteristics and liver volumetry. (XLSX)

## Abbreviations

CT: computed tomography
FLR: future liver remnant
HCC: hepatocellular carcinoma
INR: International Normalized Ratio
LLV: lobar liver volumes
ns: not significant
RE: radioembolization
rLLV: relative lobar liver volumes
